# Update on the Pathogenesis, Virulence, and Treatment of *Candida auris*

**DOI:** 10.20411/pai.v7i2.535

**Published:** 2022-10-21

**Authors:** Richard R. Watkins, Rachael Gowen, Michail S. Lionakis, Mahmoud Ghannoum

**Affiliations:** 1 Department of Medicine, Division of Infectious Diseases, Northeast Ohio Medical University, Rootstown, Ohio; 2 Center for Medical Mycology, Department of Dermatology, Case Western Reserve University, Cleveland, Ohio; 3 Fungal Pathogenesis Section, Laboratory of Clinical Immunology and Microbiology, National Institute of Allergy & Infectious Diseases, National Institutes of Health, Bethesda, Maryland; 4 University Hospitals Cleveland Medical Center, Cleveland, Ohio

**Keywords:** *Candida auris*, virulence, antifungal resistance, fungemia, immune responses

## Abstract

*Candida auris* is an emerging, multidrug resistant fungal pathogen that causes considerable morbidity and mortality. First identified in Japan in 2009, it has since been reported in more than 40 countries. *C. auris* can persist for long periods on different environmental surfaces as well as the skin. Clinical isolates are typically resistant to commonly prescribed antifungal drugs. Increasingly recognized as a cause of infections and outbreaks in nosocomial settings, *C. auris* is difficult to identify using traditional microbiological methods. One of the main reasons for the ongoing spread of *C. auris* is the multitude of virulence factors it possesses and uses against its human host that enables fungal persistence on the skin surface. Yet, many of the virulence mechanismsare unknown or remain incompletely understood. In this review, we summarize the evolution of virulence of *C. auris*, offer recommendations for combating this important human pathogen, and suggest directions for further research.

## INTRODUCTION

**Figure 1.**
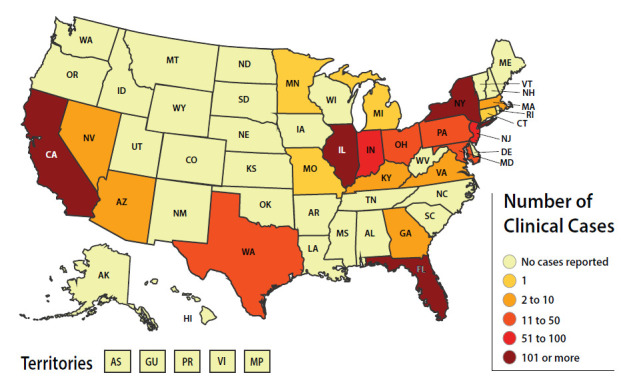
Reported clinical cases of *Candida auris* in the United States, January 01, 2021 to December 31, 2021.

Source: https://www.cdc.gov/fungal/candida-auris/tracking-c-auris.html#historical

The emergence of *Candida auris* as a human pathogen has been met with grave concern from clinicians and public health authorities. Since it was first identified in Japan in 2009, *C. auris* has spread rapidly worldwide [1]. *C. auris* became a nationally notifiable pathogen in the United States in 2018. The Centers for Disease Control and Prevention (CDC) subsequently designated *C. auris* an urgent threat due to its resistance to antifungal therapies [2]. As demonstrated in Figure 1, nearly half of all US states reported at least 1 case in 2021. *C. auris* has evolved a number of novel features that mediate environmental adaptation, host survival, and pathogenicity, which are unique or otherwise similar to other *Candida* species [3]. Through the use of whole genome sequencing (WGS), 5 different clades based on geography have been recognized: South Asian clade I, East Asian clade II, African clade III, South American clade IV, and clade V from Iran. The clades differ in their antifungal resistance profiles, with clade II exhibiting less resistance than the others, and in their propensity to persist on mammalian skin [4].

The fungal species most similar to *C. auris* is *C. haemulonii*, another phylogenetically related drug-resistant *Candida* species that is also being increasingly reported from health care facilities [5]. An ascomycetous fungus that grows as yeast, *C. auris* forms shiny, smooth, whitish colonies on fungal growth media. The name ‘auris' comes from the Latin word for ear because it was first isolated from the ear canal of a hospitalized patient. The overuse of antifungal agents has been hypothesized as the reason *C. auris* has become a human pathogen after previously living harmlessly in the environment [6]. Another hypothesis regarding the pathogenicity of *C. auris* is that it has evolved due to thermal adaptation from climate change [7].

## IDENTIFICATION CHALLENGES

*C. auris* is often incorrectly identified in the microbiology laboratory when traditional testing methods are employed. It is frequently misidentified as *C. haemulonii* by the commercial identification system VITEK (bioMérieux, Marcy l'Étoile, France) and as *Rhodotorula glutinis* by the API-20C AUX (bioMérieux) [8]. These systems employ precast panels of assimilation/growth tests using sets of carbon and nitrogen compounds and are widely used for routine identification of yeast in clinical microbiology laboratories. Many common biochemical tests also misidentify *C. auris*, most often as *R. glutinis* or *C. haemulonii* [9]. Mizusawa *et al* reported that all *C. auris* isolates were misidentified as *Rhodotorula glutinis* by API 20C AUX (bioMérieux); *C. haemulonii* (except one as *C. catenulata*) by Becton Dickinson Phoenix (BD Diagnostics, Sparks, MD); *C. haemulonii* by VITEK 2 (bioMérieux); and *C. famata, C. lusitaniae, C. guilliermondii*, or *C. parapsilosis* by MicroScan (Beckman Coulter, Pasadena, CA) [10]. Matrix-assisted laser desorption ionization-time of flight mass spectrometry (MALDI-TOF MS) is more accurate compared to the other methods for identifying *C. auris* and allows for subsequent epidemiological characterization of strains [11].

Recently, new formulations of chromogenic media have been specifically developed for identifying *C. auris*, including CHROMagar *Candida* Plus (CHROMagar, France) [12] and HiCrome *C. auris* MDR selective agar (HiMedia, Mumbai, India) [13]. On CHROMagar Candida Plus, *C. auris* forms characteristic white colonies with blue-green halos that are more evident after 72 hours of incubation at 35°C than after 48 hours. However, distinguishing between closely related species including *C. haemulonii, C. pseudohaemulonii*, and *C. duobushaemulonii* requires using additional parameters besides color, including colony size and ability to grow at 35°C. Other identification methods include molecular tests like DNA sequencing and WGS [14]. Future approaches are likely to incorporate machine-learning algorithms with routine microbiological testing [15].

## RESISTANCE ATTRIBUTES

Resistance to antifungal drugs by *C. auris* is well-documented and is one of the main drivers of its pathogenicity. *C. auris* exhibits consistently high fluconazole minimal inhibitory concentrations (MICs) and variable susceptibility to the other triazoles, echinocandins, and amphotericin B [16]. Indeed, *C. auris* is the first *Candida* species to be classified as multidrug resistant (MDR). Of even greater concern is the emergence of *C. auris* strains that are pan-resistant, although novel antifungals such as ibrexafungerp and fosmanogepix have demonstrated *in vitro* and *in vivo* efficacy against *C. auris* [17]. Ostrowsky *et al* described 3 patients with pan-resistant *C. auris* that developed after receiving antifungal medications, including echinocandins [18]. The isolates were initially susceptible to echinocandins; resistance was detected after treatment, indicating that it emerged during therapy. There was no evidence of transmission of the resistant isolates following the initial infections. Thus, patients on antifungal treatment for *C. auris* should be closely monitored for clinical improvement. Follow-up cultures are recommended, and repeat susceptibility testing should also be conducted, especially for patients who received echinocandins.

## CLINICAL CONSIDERATIONS AND RISK FACTORS FOR INFECTION

The clinical presentation of *C. auris* is often non-specific and similar to other types of systemic infections [19]. Most of the reported cases have been associated with invasive infections, such as candidemia and infected devices. The majority of *C. auris* infections occur in adults, and there is a propensity towards critically ill patients in intensive care units (ICUs). The mortality rate for *C. auris* candidemia is high and ranges from 30% to 72% [19]. Because *C. auris*, like other *Candida* species, can be a colonizer of non-sterile body sites (eg, lungs, urine, and skin), it is important to ascertain the signs and symptoms of infection when *C. auris* is identified in clinical specimens. In addition to candidemia, *C. auris* has been reported to cause urinary tract infections (UTIs), otitis, wound infections, skin abscesses (often related to catheters), myocarditis, meningitis, and osteomyelitis [20].

The COVID-19 pandemic has led to a large number of critically ill patients admitted to ICUs. Not surprisingly, outbreaks of SARS-CoV-2 and *C. auris* co-infections have been reported from a number of facilities [21, 22]. A meta-analysis from studies published during the first 18 months of the pandemic found an increased prevalence of antimicrobial resistance (AMR) in patients with COVID-19 who subsequently developed bacterial and fungal infections, including a high prevalence of MDR *C. auris* [23]. The majority of hospitalized patients with COVID-19 received at least one antibiotic (often azithromycin), a known risk factor for nosocomial fungal infections. Hospital admission, especially to the ICU, also increases the risk of patients with COVID-19 becoming colonized with *C. auris*, who can then serve as reservoirs for transmission to other patients and the environment. Using WGS, Yadav *et al* found that 10% of fomite samples contained *C. auris* in rooms about 8½ days after *C. auris* colonized patients were admitted [24]. The high effectiveness of 1% sodium hypochlorite against *C. auris* was noted, thus providing a potential method for reducing its transmission in nosocomial settings. While it is possible for any individual to become infected with *C. auris* regardless of health status, a number of risk factors have been identified (Table 1). Pandya *et al* found the most common age range for *C. auris* infections was 61 to 70 years [25]. Skin colonization with *C. auris* also increases the risk for disseminated infection, particularly candidemia [26, 27]. The skin of many nursing home residents is chronically colonized with *C. auris*, which likely represents an important reservoir for its ongoing spread [28].

**Table 1.** Risk Factors for *Candida auris* Infection

Urinary catheters Central venous catheters Malignancy Chronic kidney disease Neutropenia Total parenteral nutrition (TPN) Increased hospital LOS Mechanical ventilation Immune compromise Recent (ie, within previous 90 days) or ongoing broad-spectrum antibiotic use Surgical procedures HIV/AIDS

LOS: length of stay

## VIRULENCE FACTORS AND HOST RESPONSE

Virulence factors are defined as genes and other advantages that contribute to the pathogenicity of an organism. These factors can be maintained within the genome on pathogenicity islands; these are areas containing segments of genes that directly confer infectious qualities leading to disease in the host [29]. Often these genetic advantages promote pathogen survival in otherwise inhospitable environments, allowing the organism to spread and multiply. In the case of *Candida* species, *C. albicans* is considered a model study organism due to the decades of research analyzing its contribution to systemic and vaginal infections. For example, a well-studied *C. albicans* virulence factor is PHR1, a gene that when expressed allows the yeast to survive in neutral pH environments such as the bloodstream or tissues [30]. In contrast, the RPH2 gene promotes survival in acidic environments such as the vaginal canal. Other *Candida* species such as *C. dubliniensis* maintain homologues to these genes; PHR1 and PHR2 serve similar functions for this organism as well [31]. *C. maltosa* also has homologues EPD1 and EPD2 that function similarly to PHR1 of *C. albicans.*

The *Candida* genus also displays some species that are able to change phenotype and morphology, typically called phenotypic switching [32]. When plated on agar, *C. albicans* can switch from white colonies into a filamentous form. *In vivo*, these hyphae protrude and force their way into skin layers, gastrointestinal tract, or other boundaries not otherwise able to be accessed by the yeast [32]. When *C. auris* was first studied for phenotype switching, it did not produce hyphae or pseudohyphae. Yet under high salt conditions and in biofilm formation, *C. auris* can produce basic pseudohyphae [33]. It should be noted, however, that *C. auris* lacks candidalysin (ECE1) and hyphal cell wall protein (HWP1), which are essential for full hyphal growth [34].

Perhaps the largest contributing factor to virulence of *Candida* lies with secreted aspartyl protein-ases (SAPS). These enzymes have been implicated in many functions such as adhesion, biofilm, cell-wall formation, and host tissue degradation [35]. SAPS have also been shown to downregulate the immune complement system and evade the immune system altogether via preventing inflammatory responses [36]. *C. albicans* employs 10 genes encoding SAPS, of which SAP4, SAP5, and SAP6 are the most important for virulence [37]. In *C. auris,* hydrolases are the predominant secreted enzyme, comprising 42% of their encoded enzymes. Orthologous genes for 4 SAPS have also been identified within the *C. auris* genome [38]. In one study, *C. albicans* SAPS were most active at 25°C, 37°C, and 40°C [39]. In comparison, *C. auris* SAPS were elevated at 42°C, indicating that the yeast might have increased survivability at higher temperatures than other *Candida* species.

Beyond SAPS, lipases are another group of virulence-implicated enzymes that can contribute to biofilm formation, damage to host cells, and evasion of the immune system [40]. When lipases were inhibited via knock-down experiments, as seen with a *C. parapsilosis* experiment, the yeast could not effectively evade immune cells and were increasingly taken up by macrophages as compared to the control yeast strains [41]. In addition, *C. albicans* and *C. parapsilosis* mutants lacking lipases were less virulent in a neonatal rat model [42]. *C. auris* is able to secrete phospholipases as well, though this ability is strain-dependent and only present in up to 37.5% of isolates [43]. In comparison, 64% of *C. auris* strains can produce proteinases [43]. Furthermore, *C. auris* phospholipases are largely weaker compared to *C. albicans* phospholipases, with the exception of the CBS 12770 *C. auris* strain [43].

Another key virulence factor is the ability to form biofilms. Through transcriptomic analysis, it was found that *C. auris* upregulates adhesin proteins CSA1, IFF4, PGA26, and PGA52 when forming and maintaining biofilms [44]. As the biofilm matures, ABC transporter proteins such as CDR1, SNQ2, and YHD3 activate, and major facilitator superfamily proteins MDR1 and RDC3 are upregulated [44]. Also, adherence factors allow for cell-cell adhesion within the biofilm. Agglutinin-like sequence (ALS) proteins ALS1 and ALS5 of *C. auris* are hypothesized to contribute to adherence of biofilm formation in *C. auris* according to transcriptomic analyses [44]. Interestingly, *C. auris* retains a much lower amount of ALS and other adhesin genes when compared to *C. albicans,* which may help explain why *C. albicans* biofilms are more common and robust [38].

*C. auris* exhibits unique evasion mechanisms to host immune systems. An *in vitro* model utilizing human neutrophils co-cultured with *C. auris* did not inhibit fungal growth: indeed, *C. auris* grew past the initial inoculum amount [45]. Compared to *C. albicans,* when the experiment was repeated, neutrophils decreased fungal growth by 75%. When *C. albicans* and *C. auris* were co-cultured *in vitro* with human neutrophils, neutrophils primarily targeted and engulfed *C. albicans* rather than *C. auris* cells [45]. To further explore this mechanism of action, an *in vivo* zebrafish model was assessed for *C. auris* evasion of neutrophils. In this animal model, *C. albicans* only maintained a 5% survival rate, whereas *C. auris* appeared much more resilient to neutrophils. When an *in vivo* sample was cultured, it was found that the host immune system recruited 50% fewer neutrophils for *C. auris* than *C. albicans.* Further fluorescent microscopy of the neutrophils from this experiment revealed that neutrophils did not form neutrophil extracellular traps (NETs) when exposed to *C. auris in vivo* [45]. This indicates that perhaps *C. auris* has a potent inhibitory effect on neutrophils to avoid innate immune detection, specifically targeting NETS to avoid being engulfed.

To further test host immune response to *C. auris* bloodstream infection, another study utilized immunocompetent C57BL/6 mice [46]. Mice were infected with *C. auris* and splenocytes were isolated from surviving mice after 7 days. Flow cytometry was then used to determine levels of immune signaling molecules: programmed cell death protein (PD-1) and cytotoxic T-lymphocyte-associated protein 4 (CTLA-4) were measured on the surface of T cells, natural killer cells, macrophages, and natural killer T cells. Results from the flow cytometry revealed PD-1 expression was elevated, especially on macrophages from the spleen. Spearman's rank correlation coefficient was P = 0.95 when calculated for fungal burden and PD-L1 expressing macrophages. This means that as the growth of systemic *C. auris* increases, so too does PD-1 signaling. Since PD-1 is considered an inhibitor of T-cell activation, this study provides evidence of a *C. auris* immune evasion mechanism [47]. Due to these results, there is potential that *C. auris* interacts with or stimulates PD-1 expression, which will have downstream effects that inhibit T-cell activation and proper immune response [47]. This observation may have therapeutic implications, since antibodies against PD-1 induction would prevent this inhibition of T-cell activation and counteract this immune evasion strategy. However, more research is needed on the exact mechanism that *C. auris* employs to increase PD-1 expression and evade the immune system.

## GENOMICS

Before its discovery in 2009, *C. auris* had been largely misidentified as *Candida haemulonii*. Using genetic sequencing mechanisms, Vallabhaneni *et al* distinguished this emerging yeast from other *Candidal* species such as *C. haemulonii* [48]. While much of the entire *C. auris* genome remains unknown, there have been several studies to determine phylogenetic relationships, clade categorization, and virulence factors [49] [50].

Using Illumina and Nanopore sequencing, 7 chromosomes have been identified belonging to *C. auris* [51]. The pathogen's genome also shares more homology with that of *C. haemulonii* than *C. albicans* and is most closely linked phylogenetically to *C. heveicola* [51]. It is thought that chromosomal rearrangement may have contributed greatly to the emergence of the 5 *C. auris* clades: clade I and clade III have translocated DNA from prominent isolates B8441 and B11221, respectively [34]. Despite their genetic divergences, the 4 main clades of *C. auris* share 98.7% genetic similarity.

Much of the *C. auris* genome has been uncovered via genetic homology to other candidal organisms and while there are several similarities, *C. auris* also diverges from common reference species such as *C. albicans.* For example, through genetic comparison, highly expressed *C. albicans* virulence genes ECE1 (candidalysin) and HWP1 (hyphal cell wall protein) were found not to be conserved in the *C. auris* genome [33]. In *C. albicans,* the HWP1 gene is implicated in hyphal development, a feature which improves survival under high-salinity conditions [34]. It is currently unknown why *C. auris* does not have these survival genes maintained in its genome. However, the *C. auris* genome does contain several lytic enzymes to improve pathogenesis, such as hydrolases, transferases, and oxidoreductases, though the expression of these enzymes is often strain-dependent [38]. It should be noted that this enzymatic expression was found to be tantamount to other *Candida* species such as *C. albicans* and *C*. *dubliniensis* [34].

*C. auris* may however have an advantage over *C. albicans* in terms of iron acquisition and temperature tolerance. Most *C. auris* strains have active hemolysin enzymes that help competitively sequester iron for faster growth and increased spread [52]. *C. auris* also responds more aggressively to temperature stress than *C. albicans*: when compared at 42°C, *C. auris* secreted higher levels of protective aspartyl proteinase compared to *C. albicans* [39]. Currently, however, the whole *C. auris* genome has not been fully mapped; many protein sequences have been documented on public databases such as NCBI, but their functions remain unknown.

## BIOFILM

As previously mentioned, *C. auris* displays a formidable ability to generate biofilms, wherein the yeast secretes mannan-glucan polysaccharides to form a barrier to the external environment [50]. *C. auris* cells anchor themselves to a surface via adhesin proteins to initiate a biofilm state. Once tethered, the layers of biofilm thicken to form an impenetrable barrier from the immune system and antifungal therapeutics. Studies have also shown that genes controlling efflux pumps are upregulated during biofilm formation, perhaps aiding in ejecting antifungal agents [50]. When compared to *C. albicans, C. auris* has a decreased propensity for biofilm formation and adherence (P = 0.01) [43].

However, specific *C. auris* strains may show a higher propensity for biofilm generation, with clade III more commonly forming aggregates leading to biofilm formation [50]. This clade maintains a higher expression of adhesins and upregulation of adhesin genes to achieve this protective barrier. The age of biofilms also contributes to antifungal resistance, since mature biofilms have shown more pan-resistance to all 3 classes (azoles, echinocandins, and polyenes) of antifungals [53]. Inhibition of biofilm efflux pumps restored susceptibility to fluconazole in one study, exemplifying the ability of mannan and glucan to otherwise sequester azole antifungals [53].

Biofilms have also proven to be a contributing factor to nosocomial spread in multiple case studies. Indeed, health care professionals are largely implicated as vectors for *C. auris* via provider-patient contact through skin colonization or surface contamination [54]. *Candida* species were found on 36.4% of hospital floors surveyed, 23.5% of sink drains, and 9.1% of all high-touch surfaces [54]. In another study, *C. auris* was allowed to grow biofilms and was tested against 13 commonly used hospital disinfectants. Overall, 50% of the products failed to prevent cell viability, 58% did not prevent transfer of *C. auris,* and 75% of the disinfectants could not prevent biofilm regrowth [55].

Biofilms therefore pose a large threat to proper sanitation in hospital settings. In a study on the effectiveness of 9 disinfectants against several *Candida* species, common surface cleaning chemicals such as Purell, Lysol, and Virex had little-to-no effect on *C. albicans, C. glabrata,* and *C. auris* growth [56]. Only bleach agents such as Clorox, OxyCide, and Oxivir TB reduced colony formation significantly [56]. Unfortunately, ammonium-based cleaning agents are widely used across hospitals, even though they have proven less effective than bleach [56]. It has thus been proposed that alternative strategies may be required or used in tandem with cleaning agents. In lieu of this dilemma, photodynamic therapy has been studied to avoid antifungal resistance and the additional protection offered by biofilms. Certain wavelengths of light may be able to bypass the protective biofilm layer and penetrate pathogenic cells, if used in tandem with photosensitizing compounds and molecular oxygen [57]. The ability of light to target multiple cellular components also decreases the chance of developing resistance mechanisms [57].

In one such study investigating the ability of UV light to penetrate *C. auris* colonization, a decontamination device emitting 254 nm of UV-C light was assessed for germicidal properties. Notably, a 10-minute exposure time did not reduce *C. auris* growth as much as *C. glabrata* and *C. albicans*, further attesting to the robust nature of this pathogenic organism [58]. An exposure time of 20 to 30 minutes was required to significantly reduce growth of *C. auris* [56]. Additionally, the diameter of the spread of *C. auris* contributed to colony formation under UV light. *C. auris* was tested for colony reduction at spreads of 10 mm, 20 mm, and 40 mm diameter. When exposed to UV light at a distance of 5 feet, colonies with a larger surface area of 40 mm were more effectively reduced than at 10 mm [58]. Thus, it was hypothesized that the larger cell size of *C. auris* in comparison to bacteria and the surface area of the pathogen may contribute to the longer exposure time, as it may take longer for the UV light to properly penetrate cells underneath a protective biofilm layer [58]. Photodynamic therapy therefore represents a promising alternative to clinical antifungals that may overcome resistance mechanisms due to biofilm.

### Animal Models for Evaluating Activity Against Antifungal Agents

*C. auris* uniquely colonizes host skin externally as well as systemically, causing infections that can enter the bloodstream and spread throughout the body. This invasive form of candidiasis is recognized as candidemia and can often prove more fatal than cutaneous infections [59]. Fortunately, mucocutaneous infections of *Candida* species are typically more common, though it should be recognized that vulnerable immunocompromised and otherwise sick individuals in nosocomial settings are most susceptible and at risk [59]. This fact alone creates urgency for better antimicrobial cleaning agents in hospitals to avoid nosocomial spread among patients, as well as antifungals that are effective against systemic infections caused by *C. auris*.

There are currently several animal models for evaluating *C. auris* antifungal susceptibility, mainly utilizing small mammalian species such as mouse or guinea pig. One such process involves *in vivo* cutaneous infection of guinea pig or mouse, in which the animal hair is shaved to better evaluate skin infection of the yeast [60]. Skin samples from sacrificed animals can then be assessed for colony formation through plating on nutrient-rich media. Colonies are then counted to evaluate growth and cutaneous spread is visually assessed on the shaved animal [60]. This model is often utilized to determine the pathogenicity of various *C. auris* strains, as well as host response to skin colonization and therapeutic approaches to decolonize the skin.

This model can also be utilized to test novel antifungal agents against cutaneous infection. The most effective strategy for evaluating new antifungal agents is through susceptibility testing. This process involves initially evaluating the MIC. Standard MIC values for a candidal organism can be found on the ARTEMIS Global Antifungal Surveillance Program database, which holds information for about 5,346 *Candida* isolates [61]. This compiled information gives a good baseline for susceptibility testing for both the species and type of drug administered. Though MIC values are technically arbitrary, they give a good sense of comparison especially when testing novel antifungals. Susceptibility testing can be achieved by making serial dilutions of the drug and exposing the organism of interest to varying concentrations [60]. A positive finding of the *in vitro* testing provides rationale for moving candidate compounds forward for *in vivo* evaluation, as well as provides dosage guidance.

Animal models are also useful for measuring and assessing systemic host response to the pathogen. In one such study, skin colonization of *C. auris* was found to reside in deeper tissue, leading to invasive candidemia [62]. Innate and adaptive immune responses were then evaluated as a model for host defense. Both CD4+ and CD8+ T cells were found on the skin surface in large quantities, with helper CD4+ Th17 cells accumulating in deeper skin layers [62]. Studies such as these were crucial to discovering the prominent role of IL-17 in cutaneous and subcutaneous *C. auris* infection. Indeed, IL-17 deficient mice do not mount as protective a response against cutaneous *C. auris*, indicating that IL-17 is crucial for immune detection and response to this fungal pathogen [62].

Mouse models can also provide an estimate of virulence for *C. auris* when compared to other *Candida* species. Invasive *C. albicans,* for example, has been well-documented in scientific literature for decades, and thus provides a good baseline model for novel fungal pathogens. In one such study, *C. auris* was compared with *C. haemulonii, C. glabrata,* and *C. albicans in vivo* [63]. One (1) × 10^5^ colony forming units (CFUs)/mouse inoculum was injected into the tail vein of healthy female mice that were then monitored for up to 30 days. After sacrificing, mouse kidney, liver, spleen, and lung were aseptically harvested and assessed for fungal burden. Notably, the median survival time (MST) of *C. auris-*infected mice was 16 to 17 days, with roughly a 30%-40% survival rate. This was comparable to *C. albicans* MST of 13 days and survival rate of 20%. For reference, *C. glabrata* obtained an MST of 19 days with 30% survival, while *C. haemulonii* had 100% survival.

Beyond murine models, some researchers have opted for fly models to test virulence and anti-fungal treatments. In 2019, one research group posited that the species *Drosophila melanogaster* could be used as a model of *C. auris* infection [46]. Previous *D. melanogaster* models had shown promise with *Aspergillus* fungal strains [64]. Flies used in this study were Toll deficient, providing a streamlined, immune deficient response to infection. Flies could also be bred and ready for infection in a mere 3 weeks, creating a time-efficient animal model for fungal infection.

The 2019 study with *C. auris* then utilized female flies infected with *C. auris,* using *C. albicans* as a control [46]. Though *in vitro* growth of *C. auris* was comparably slower than *C. albicans, C. auris* infection proved more fatal, with over 80% of *C. auris* infected flies dying before the 7-day mark, compared to 67% of *C. albicans* infected flies. This finding correlates with expected pathogenicity of *C. auris*, supporting the idea that fly models may be useful for evaluating the virulence as well as the antifungal activity of candidate antifungal compounds. Furthermore, assessment of fungal burden proved simple in this experimental model: flies can be sacrificed through freezing and then directly homogenized in a tube by vortexing. The resulting mixture can then be directly plated on agar and grown for colony counting.

Overall, animal models are vital for understanding this novel pathogen and modeling its route of infection. Utilizing both murine and insect models can give unique insights into host response and provide unique benefits in the form of cost and time efficiency. Ultimately, much is still unknown about *C. auris,* but maintaining multiple forms of animal models provides the tools necessary to uncover these mysteries.

### Resistance to Antifungal Agents

In cases of fungal infection, 3 main classes of antifungal medication are often administered for clinical treatment: azoles (eg, fluconazole), polyenes (eg, amphotericin B), and echinocandins (eg, caspofungin) [65]. The CDC currently estimates that 90% of *C. auris* strains maintain resistance to fluconazole, 30% to amphotericin B, and 5% to echinocandins [65]. There are several factors that have been identified to contribute to antifungal resistance of *C. auris*, including biofilm formation, efflux pumps, genetic predisposition, and cladal phylogeny. Often multiple factors cooperate in tandem to increase overall resistance for the organism.

First in the arsenal of this pathogenic yeast are efflux pumps, which are transport channels within the cell membrane that eject molecules that may threaten survival. Of note, *C. auris* possesses 2 major efflux pumps implicated in antifungal resistance: the ATP Binding Cassette (ABC) and Major Facilitator Superfamiliy (MFS) transporters [65]. These pumps are most often implicated in azole resistance and were first identified due to their homology to *C. albicans* efflux pump genes [65]. The *C. auris* gene CDR1 was identified as the ortholog for ABC transporters, and proof-of-function was determined via gene knockout, wherein 2 independent studies found that CDR1 removal increased azole susceptibility of the yeast [66].

A second gene of interest in terms of antifungal resistance is ERG11, an ergosterol gene crucial to *C. auris* cell membrane development. ERG11 specifically encodes the enzyme lanosterol 14-alpha-demethylase, which converts lanosterol into ergosterol for cell membrane structure and integrity [67]. Mutations to ERG11 are correlated with increased azole resistance, with 3 common “hot spot” regions in the gene that are most often mutated. *C. auris* strains with changes to Y132F or K143R are the most implicated as responsible for the increased resistance. Studies analyzing this mutation indicated that strains with Y132F or K143R substitutions caused resistance to double in *C. auris* strains when compared to a *Saccharomyces cerevisiae* control [68].

Another gene of interest in *C. auris* antifungal resistance is FKS, wherein FKS1 and FKS2 encode 2 subunits of a β (1,3) D-glucan synthase [69]. This enzyme is crucial for the biosynthesized glucan components of the fungal cell wall. Echinocandin antifungals target this enzyme to prevent cell wall regeneration and formation, effectively creating holes in the membrane leading to fungal mortality. Furthermore, prevention of new glucan deposition in the fungal membrane inhibits growth of the pathogen. Mutations to the FKS genes, specifically an S639F amino acid substitution, have been shown to drastically increase overall echinocandin resistance [65].

Beyond mutations, *C. auris* also employs a common bacterial and fungal strategy for survival: the formation of biofilms. Within a standard biofilm, there is a layer of fixed sessile cells that tightly adhere to one another, and a separate layer of planktonic surface cells capable of breaking free from the biofilm, allowing the spread of infection [53]. *C. auris* is capable of growing both cell types, with planktonic cells living freely in suspension and adherent cells forming a biofilm, and thus antifungals may have a hard time working effectively against both forms. There is also the added issue of biofilm secretions, a mixture of glucan and mannan polysaccharides, protein, and DNA that form a protective barrier from antifungals [70]. In this way, biofilms act as a beneficial community providing both a shield and a genetically varied ecosystem as protection for the organism.

*C. auris* strains growing as biofilms can also maintain several unique characteristics, owing to the variation of genetic material within the community. For example, cells within the biofilm with upregulated efflux pump genes will help shield other cells from azole penetration. Studies have indicated that *C. auris* living in biofilms exhibit increased function of ABC and MFS transporters, which increases the overall antifungal resistance of the cellular community [53]. Additionally, it can be inferred that any cells containing the necessary FKS or ERG11 mutations would also provide protection against other classes of antifungals. Therefore, biofilms provide an extra layer of defense in combination with other mutations that may confer resistance.

Unfortunately, biofilms also contribute to nosocomial infection. With the physical protective layer of polysaccharides provided by the collective biofilm, colonies may be able to survive in environments such as hospital beds, catheters, hospital trays, sinks, and the surface of human skin [53]. This may afford cells protection from any conditions of fomites that may otherwise be inhospitable, such as temperature, material, pH, chemical treatments, etc. This circumstance is especially concerning as immune compromised or otherwise sick individuals within hospital settings may be even more susceptible to *C. auris* infection. Particularly, immunosuppressed individuals may be more prone to severe candidemia and thus the infection would increase fatality compared to infections in an immunocompetent patient.

There is also a correlation between *C. auris* clade and antifungal resistance mechanisms. As mentioned previously, certain clades exhibit different levels of antifungal susceptibility. For example, research on the South African clade (clade III) demonstrates high azole resistance, but lower polyene and echinocandin resistance [71]. In this particular study, 12 out of 13 clade I isolates were resistant to both fluconazole and amphotericin B. All 13 of these samples also maintained ERG11 mutations at the Y123F site [71], though it should be noted that 13 isolates are a small sample size to generalize overall resistance of the clade as 100%.

With all of these contributing factors strengthening antifungal resistance, researchers have had to employ new strategies to fight this tenacious pathogen. One such technique involves multi-drug treatments or combination therapy, wherein synergistic effects from antifungals add the extra coverage needed to kill resistant isolates. For example, Gharehbolagh *et al* noted synergism between voriconazole and micafungin (100%), flucytosine and amphotericin B (7%), and flucytosine with micafungin (7%) [72]. However, no synergistic effects were noted with any other combination of traditional antifungals. Furthermore, this study evaluated antifungals in combination with antibiotics. Of these experiments, only caspofungin and colistin showed prominent synergism (100%), while fluconazole with miltefosine, ivermectin, or nafcillin failed to show any synergistic effects. This calls for more studies to identify combinations that have potent *C. auris* inhibitory activity.

One last technique is currently under evaluation as an effective antifungal treatment: the combination of a novel drug with photodynamic therapy. As previously mentioned, photodynamic therapy may provide a way to inhibit biofilms without the added concern of developing antifungal resistance. This novel form of treatment has been researched in tandem with a novel antifungal agent called ibrexafungerp, which has shown the ability to overcome resistant strains of *C. auris,* as well as other *Candida* species. The mechanism of action of ibrexafungerp targets glucan synthesis of the cell wall, specifically inhibiting β-(1,3)-D-glucan development, much like echinocandins, even though it has subtle differences between it and traditional candins [73]. There is *in vitro* evidence in these drug trials that ibrexafungerp can act on biofilms, azole-resistant strains, echinocandin-resistant strains, as well as *Aspergillus* and non-mold, pathogenic fungi [74]. This pharmaceutical looks very promising for treating clinical candidiasis, and ibrexafungerp was recently approved by the FDA for the treatment of vulvovaginal candidiasis and will continue to be evaluated for other diseases [73].

## CONCLUSIONS

The emergence of *C. auris* as a human pathogen is a serious threat to public health. Since *C. auris* was first identified, a considerable amount of scientific investigation has led to important discoveries regarding its pathogenesis and virulence mechanisms. However, further research is necessary to understand the global epidemiology of multidrug-resistant *C. auris* infections and to elucidate additional risk factors, modes of transmission, novel therapeutic options, and effective environmental cleaning and disinfection methods against *C. auris* in order to improve patient outcomes.
